# Beta-Site Amyloid Precursor Protein-Cleaving Enzyme Inhibition Partly Restores Sevoflurane-Induced Deficits on Synaptic Plasticity and Spine Loss

**DOI:** 10.3390/ijms23126637

**Published:** 2022-06-14

**Authors:** Xingxing Wang, Qinfang Shi, Arpit Kumar Pradhan, Laura Ziegon, Martin Schlegel, Gerhard Rammes

**Affiliations:** Department of Anesthesiology and Intensive Care Medicine, Klinikum Rechts der Isar, Technical University of Munich, 81675 Munich, Germany; xingxing.wang@tum.de (X.W.); qinfang.shi@tum.de (Q.S.); arpit.pradhan@tum.de (A.K.P.); l.ziegon@web.de (L.Z.); martin.schlegel@tum.de (M.S.)

**Keywords:** isoflurane, sevoflurane, xenon, synaptic plasticity, β-site amyloid precursor protein-cleaving enzyme, dendritic spines

## Abstract

Evidence indicates that inhalative anesthetics enhance the β-site amyloid precursor protein (APP)-cleaving enzyme (BACE) activity, increase amyloid beta 1-42 (Aβ_1–42_) aggregation, and modulate dendritic spine dynamics. However, the mechanisms of inhalative anesthetics on hippocampal dendritic spine plasticity and BACE-dependent APP processing remain unclear. In this study, hippocampal slices were incubated with equipotent isoflurane (iso), sevoflurane (sevo), or xenon (Xe) with/without pretreatment of the BACE inhibitor LY2886721 (LY). Thereafter, CA1 dendritic spine density, APP processing-related molecule expressions, nectin-3 levels, and long-term potentiation (LTP) were tested. The nectin-3 downregulation on LTP and dendritic spines were evaluated. Sevo treatment increased hippocampal mouse Aβ_1–42_ (mAβ_1–42_), abolished CA1-LTP, and decreased spine density and nectin-3 expressions in the CA1 region. Furthermore, CA1-nectin-3 knockdown blocked LTP and reduced spine density. Iso treatment decreased spine density and attenuated LTP. Although Xe blocked LTP, it did not affect spine density, mAβ_1–42_, or nectin-3. Finally, antagonizing BACE activity partly restored sevo-induced deficits. Taken together, our study suggests that sevo partly elevates BACE activity and interferes with synaptic remodeling, whereas iso mildly modulates synaptic changes in the CA1 region of the hippocampus. On the other hand, Xe does not alternate dendritic spine remodeling.

## 1. Introduction

The inhalational anesthetics (e.g., isoflurane (iso) and sevoflurane (sevo)) are widely used in clinics [1]. However, studies have reported that exposure to inhaled anesthetics may hamper synaptic plasticity [2,3,4]. For instance, the attenuation of the hippocampal long-term potentiation (LTP) has been shown in the presence of volatile anesthetics iso and sevo and the noble gas xenon (Xe) [2,5,6]. Although numerous studies have hypothesized the modulation of glutamate and γ-aminobutyric acid (GABA) receptors as possible mechanisms, the exact molecular and morphological substrates are still a matter of debate [7,8].

Dendritic spines are protrusions that receive excitatory synaptic input and trigger postsynaptic responses [9], thus providing a morphological basis for synaptic plasticity. It has been shown that inhaled anesthetics can modulate dendritic spine remodeling. In the neonatal period, iso causes age- and time-dependent changes in dendritic spines, whereas sevo turns out to have a potent bidirectional effect on spine density [10]. However, most of these studies focused on the effects of iso or sevo on the early developmental stages. The anesthetic effects on the adult hippocampus, however, remain to be further investigated [10]. Moreover, little is known about the mechanism of the gaseous anesthetic Xe on hippocampal dendritic spine plasticity.

Recent reports indicate that inhalational anesthesia may potentially aggravate or even trigger the development of Alzheimer’s disease (AD) [3,8,11]. Among the causative factors for neurodegeneration, amyloid β-protein (Aβ) accumulation is widely accepted to play a key role. Aβ is generated from amyloid precursor protein (APP) via serial proteolysis process [12,13,14] by aspartyl protease β-site APP-cleaving enzyme (BACE) and γ secretases [12]. In addition, Aβ_1–42_ has been reported to be one of the solo pathogenic forms related to synaptic loss and memory deficits [15,16,17,18].

New evidence has revealed the role of volatile anesthetics in the aberrant modulation of the APP proteolysis process [10]. In vivo and in vitro studies have reported that iso exposure induced an increase in protein levels of BACE [19,20] and mRNA levels of APP [21]. Moreover, iso evoked enhanced secretion, aggregation, and oligomerization of Aβ [19,20,21,22,23]. The accumulation of Aβ further induced a vicious cycle of apoptosis [19]. Sevo application similarly increases BACE and Aβ levels [24,25]. In addition, RNA interference-mediated silencing of BACE and APP attenuates iso-induced caspase-3, a marker for apoptosis activation [26]. However, studies are scarce regarding the modulation of BACE activity during anesthesia on hippocampal APP proteolysis procedure, physiological modulation, and morphological remodeling. Moreover, the effects of the potential neuroprotective anesthetic Xe on APP processing have not yet been evaluated.

APP also functions as a kind of cell adhesion molecule (CAM) supporting synapse formation and synaptic plasticity [14,27]. Studies have clearly demonstrated that nectin-3, a postsynaptic CAM that mediates heterophilic adhesion with presynaptic nectin-1, is essential to synaptic maintenance and hippocampus-related memory [28,29,30]. A reduction in nectin-3 in the dentate gyrus (DG) or the CA3 region evoked dendritic spine loss and memory deficits [28,29]. Whether and how the inhaled anesthetics modulate nectin-3 expression profiles are still unknown.

In the present study, we examined how exposure to equipotent concentrations of iso, sevo, and Xe might modulate APP amyloidogenic process-related proteins, nectin-3 expression, dendritic spine density, and long-term potentiation (LTP) in the CA1 region of the hippocampus. The application of a BACE inhibitor served to investigate whether the blockage of β secretase activity could reverse the detrimental consequences of anesthesia on physiological and structural abnormalities. Using a site-specific knockdown strategy to mimic reduced nectin-3 levels, we further tested the role of nectin-3 in LTP and dendritic spine plasticity in the CA1 region. By means of immunostaining, we quantified the colocalization patterns of APP and nectin-3.

## 2. Results

### 2.1. BACE Inhibition Restored the Sevo-Induced Upregulation of Mouse Aβ_1–42_ (mAβ_1–42_) Levels

**Figure 1:**
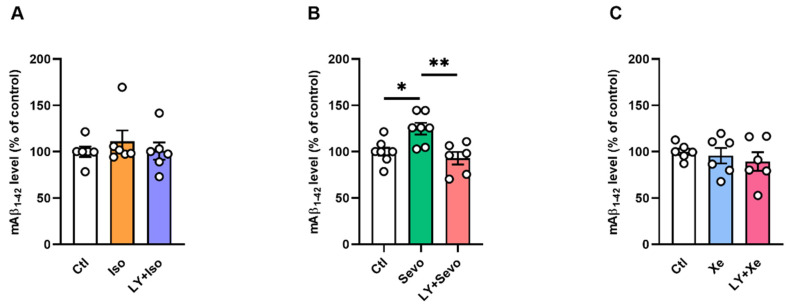
Increased mAβ_1–42_ levels by sevo were restored by BACE inhibition. (**A**,**C**) Mouse Aβ_1–42_ levels were comparable between groups. (**B**) Sevo increased mAβ_1–42_ levels dramatically (ctl vs. sevo: *p* = 0.0134), and pretreatment of LY reversed that effect (sevo vs. LY + sevo: *p* = 0.0034). One-way *ANOVA* followed by *Tukey’s* test. Data are shown as mean ± SEM. Dots represent the number of animals. Detailed statistics are provided in Table S1. * *p* < 0.05, ** *p* < 0.01. Aβ: amyloid beta, ctl: control, iso: isoflurane, LY: LY2886721, sevo: sevoflurane, Xe: xenon.

Recent studies suggest that inhalational anesthetics enhance BACE cleavage of APP and Aβ synthesis [20]. In this case, we assessed the mAβ_1–42_ levels with or without inhibition of β-secretase activity in the presence of either anesthetic. We found that, in line with previous reports [24], sevo exposure elevated hippocampal mAβ_1–42_ levels (Figure 1B, control (ctl): [100.00 ± 4.33%], sevo: [124.80 ± 6.30%], *p* = 0.0134, *Tukey’s* test of one-way *ANOVA*). Interestingly, BACE inhibition reversed the sevo-induced upregulated mAβ_1–42_ (Figure 1B, ctl: [100.00 ± 4.33%], LY + sevo: [92.90 ± 6.73%]; ctl vs. LY + sevo: *p* = 0.6633, *Tukey’s* test of one-way *ANOVA*).

To test LY2886721 (LY) alone on mAβ_1–42_ levels, we treated the slices with LY at 3 µM for 2 h. The results demonstrated that mAβ_1–42_ levels were reduced (Figure S1D, ctl [100.0 ± 0.09%] vs. LY [87.55 ± 7.72%], *U* = 0, *p* = 0.0286, two-tailed *Mann–Whitney* test, *n* = 4 mice/group). No significant changes were detected between other groups (Figure 1A, *F*_2,15_ = 0.4609, *p* = 0.6394; 1c, *F*_2,15_ = 0.4686, *p* = 0.6347, one-way *ANOVA*).

### 2.2. mAβ_1–42_ Blocked LTP and Reduced Dendritic Spine Density of CA1 Pyramidal Neurons

**Figure 2:**
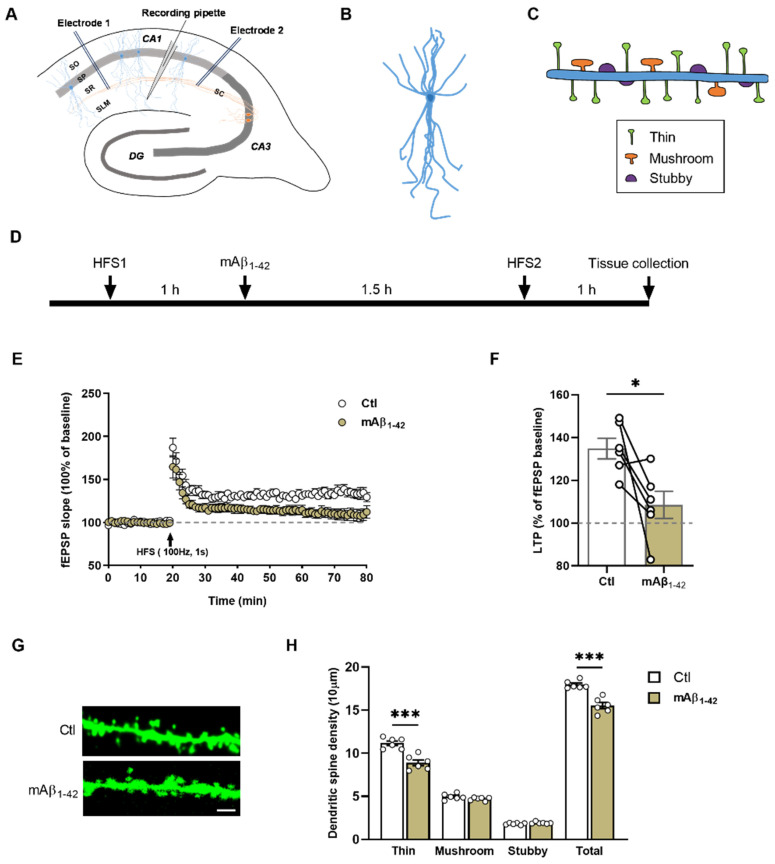
mAβ_1–42_ abolished LTP and reduced spine density. (**A**) Schematic showing the sagittal hippocampal slices. Anterograde or retrograde high-frequency stimulation (HFS, 100 Hz, 1 s) in the Schaffer collaterals pathway was triggered by two bipolar stimulation electrodes. The recording pipette was positioned in the stratum lacunosum-molecular layer of the CA1 region. (**B**) A schematic of CA1 pyramidal neuron. (**C**) Dendritic protrusions were classified and categorized as thin, stubby, and mushroom spines of CA1 pyramidal neurons. (**D**) Experimental design. (**E**) Normalized fEPSP values under control and mAβ_1–42_ application. (**F**) mAβ_1–42_ abolished LTP (*p* = 0.0081). (**G**) Representative apical dendritic segments of CA1 pyramidal neurons of both groups. Scale bar = 2 µm. (**H**) Reduced thin and total dendritic spines under mAβ_1–42_ exposure. (Thin: *p* = 0.0003; total: *p* = 0.0001). Two-tailed unpaired *t*-test. For each animal, six dendritic segments were analyzed. Data are shown as mean ± SEM. Dots in B and D represent the number of animals. Detailed statistics are provided in Table S1. * *p* < 0.05, *** *p* < 0.001. Aβ: amyloid beta, ctl: control, DG: dentate gyrus, fEPSP: field excitatory postsynaptic potentials, LTP: long-term potentiation, SLM: stratum lacunosum-moleculare, SO: stratum oriens, SP: stratum pyramidale, SR: stratum radiatum.

Our previous studies have demonstrated that human Aβ_1–42_ hampers LTP in a concentration-dependent manner [16]. In order to check if the elevated mAβ_1–42_ by sevo affects synaptic transmission and synaptic dynamics, we looked at the extracellular recordings and the impact on the dendritic spines. Incubation with 50 nM mAβ_1–42_ for 1.5 h abolished LTP (Figure 2E–F, [134.80 ± 4.82%] vs. [108.50 ± 6.37%], *t*_10_ = 3.297, *p* = 0.0081, two-tailed *t*-test, *n* = 6 mice/group). Thereafter, we quantified dendritic spines complexity in CA1 region. After 1.5 h incubation, mAβ_1–42_ decreased spine density (Figure 2G,H, [17.94 ± 0.19] vs. [15.52 ± 0.35], *t*_10_ = 6.078, *p* = 0.0001, two-tailed *t*-test, *n* = 6 mice/group), especially thin spines (Figure 2G,H, [11.17 ± 0.26] vs. [8.90 ± 0.34], *t*_10_ = 5.323, *p* = 0.0003, two-tailed *t*-test, *n* = 6 mice/group), of CA1 apical pyramidal neurons.

### 2.3. BACE Inhibition Partly Reversed the Sevo-Induced Dendritic Spines Loss

Iso and sevo have been known to reduce dendritic spine density in the hippocampus [31,32]. In this case, we further examined the impact of equipotent anesthetics on dendritic spine dynamics in CA1-pyramidal neurons.

**Figure 3:**
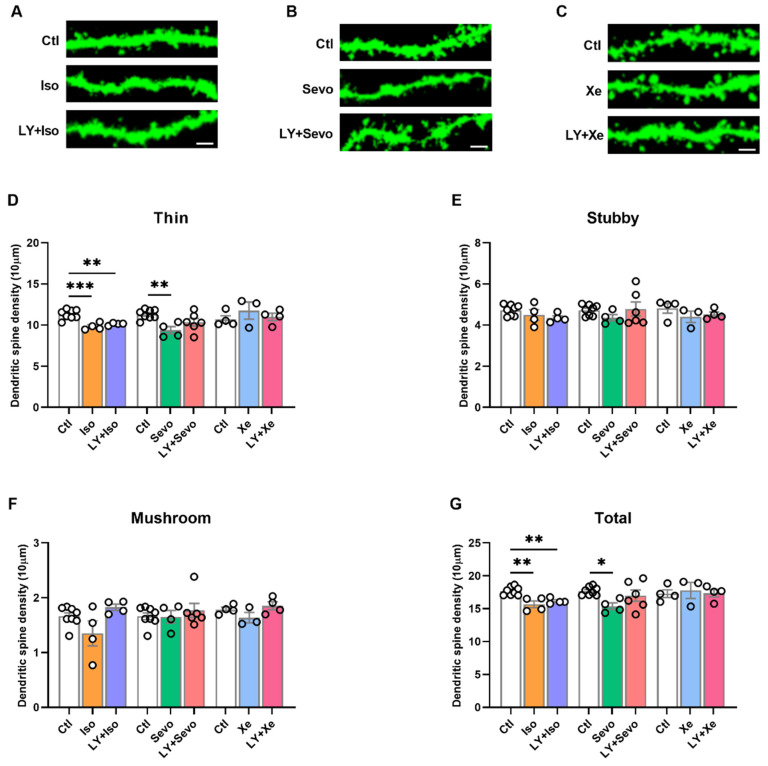
Xe did not change spine density, and BACE inhibition partly reversed the sevo-induced spine elimination, but not that induced by iso. (**A**–**C**) Representative apical dendritic segments of CA1 pyramidal neurons of all groups. Scale bars = 2 µm. (**D**) Thin spines were reduced under iso, sevo, but not Xe application (ctl vs. iso: *p* = 0.0006; ctl vs. sevo: *p* = 0.0066). (**E**,**F**) No change of stubby or mushroom spines between groups. (**G**) Total spines were significantly reduced after iso and sevo, but not Xe application (ctl vs. iso: *p* = 0.0012; ctl vs. sevo: *p* = 0.0370). LY partly restored the negative effects of sevo, but not that of iso (ctl vs. LY + iso: *p* = 0.0095; ctl vs. LY + sevo: *p* = 0.6194). Spines did not alternate under Xe or LY + Xe conditions (**D**–**G**). One-way *ANOVA* followed by *Tukey’s* test. For each animal, six dendritic segments were analyzed. Data are shown as mean ± SEM. Dots represent the number of animals. Detailed statistics are provided in Table S1. * *p* < 0.05, ** *p* < 0.01, *** *p* < 0.001. Ctl: control, iso: isoflurane, LY: LY2886721, sevo: sevoflurane, Xe: Xenon.

As shown in Figure 3G, iso evoked a reduction in thin and total spines (Figure 3D, thin spines: ctl [11.30 ± 0.20], *n* = 8; iso [9.81 ± 0.21], *n* = 4; *p* = 0.0006; Figure 3G, total spines: ctl [17.69 ± 0.23], *n* = 8; iso [15.65 ± 0.52], *n* = 4; *p* = 0.0012, post hoc with *Tukey’s* test of one-way *ANOVA*). Application of LY did not reverse the iso-induced effects on spine density (Figure 3D, thin spines: ctl [11.30 ± 0.20], *n* = 8; LY + iso [10.12 ± 0.08] *n* = 4; *p* = 0.0036; Figure 3G, total spines: ctl [17.69 ± 0.23], *n* = 8; LY + iso [16.15 ± 0.21] *n* = 4; *p* = 0.0095; post hoc with *Tukey’s* test of one-way *ANOVA*).

In the presence of sevo, thin and total spines were also reduced (Figure 3D, thin spines: ctl [11.30 ± 0.20], *n* = 8; sevo [9.37 ± 0.44], *n* = 4; *p* = 0.0066; Figure 3G, total spines: ctl [17.69 ± 0.23], *n* = 8; sevo [15.38 ± 0.49], *n* = 4; *p* = 0.0370; post hoc with *Tukey’s* test of one-way *ANOVA*). Moreover, pretreatment with LY partly reversed the sevo effects on spine dynamics (Figure 3D, thin spines: ctl [11.30 ± 0.20], *n* = 8; LY + sevo [10.34 ± 0.47] *n* = 6; *p* = 0.1351; Figure 3G, total spines: ctl [17.69 ± 0.23], *n* = 8; LY + sevo [16.99 ± 0.86] *n* = 6; ctl vs. LY + sevo: *p* = 0.6194; post hoc with *Tukey’s* test of one-way *ANOVA*).

The neuroprotective effects of Xe have been largely investigated previously [33,34,35]. Xe, at an equipotent concentration to iso and sevo, and LY + Xe exhibited no effects on spine density (Figure 3D–G, one-way *ANOVA*).

Taken together, these results suggest that Xe, in contrast to iso or sevo, does not affect spine dynamics on CA1 pyramidal neurons. Furthermore, sevo reduced spine density in CA1, which was mildly prevented by the inhibition of β-secretase activity.

### 2.4. BACE Inhibition Reversed the Sevo-Induced Reduction in Nectin-3 Levels in CA1

Cell adhesion molecules are critical in neuronal communication. Adhesion disruption may result in structural and functional imbalance leading to neurodegenerative diseases such as AD [36,37]. Postsynaptic nectin-3 mediates heterophilic adhesion with presynaptic nectin-1 and is involved in hippocampus-dependent learning and memory [28,29].

**Figure 4:**
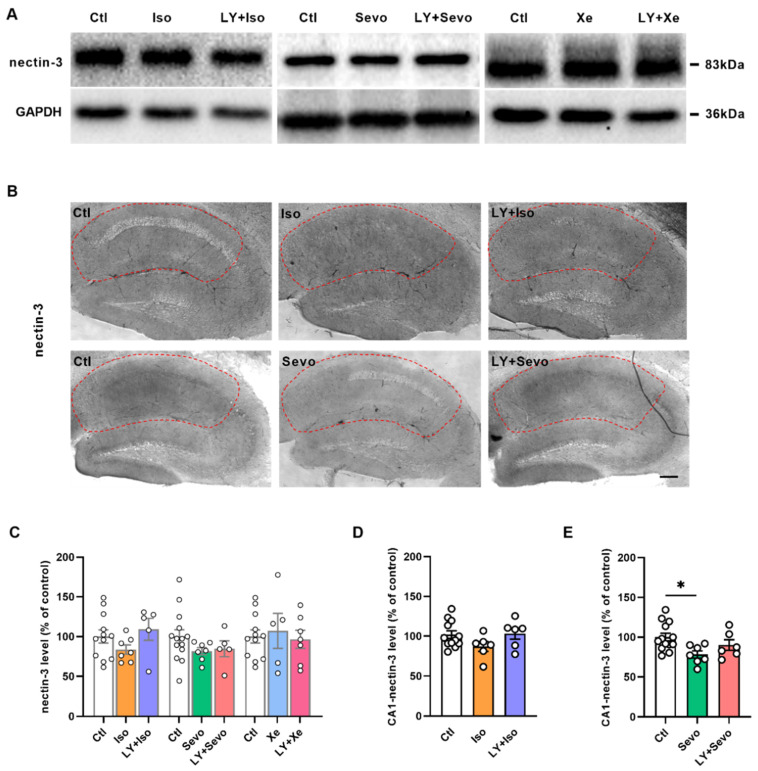
Reduced nectin-3 levels in CA1 by sevo were reversed by BACE inhibition. (**A**) Representative nectin-3 bands by Western blot. (**B**) Representative nectin-3 images by immunohistochemistry in the CA1 region (in the red circle). (**C**) Hippocampal nectin-3 levels measured by western blot were comparable between groups. (**D**) Regulation of nectin-3 levels by iso and LY + iso in CA1 region. (**E**) Sevo downregulated nectin-3 expressions in the CA1 region (ctl vs. sevo: *p* = 0.0205, *Tukey’s* test of one-way *ANOVA*), which was restored by LY + sevo. Scale bar = 200 µm. Data are shown as mean ± SEM. Dots represent the number of animals. Detailed statistics are provided in Table S1. * *p* < 0.05. Ctl: control, GAPDH: glyceraldehyde 3-phosphate dehydrogenase, iso: isoflurane, LY: LY2886721, sevo: sevoflurane, Xe: xenon.

We measured the nectin-3 levels in either the whole hippocampus or the subregions by means of Western blot and immunohistochemistry, respectively (Figure 4). Although the nectin-3 levels in the whole hippocampus were comparable between groups (Figure 4C), a reduction in nectin-3 levels was found, specifically in the CA1 region in the sevo group (Figure 4E, ctl [100.00 ± 4.86], *n* = 13; sevo [78.16 ± 4.48], *n* = 7; *p* = 0.0205, post hoc with *Tukey’s* test of one-way *ANOVA*).

### 2.5. Nectin-3 Knockdown in CA1 Region Attenuated LTP and Evoked Spine Loss

**Figure 5:**
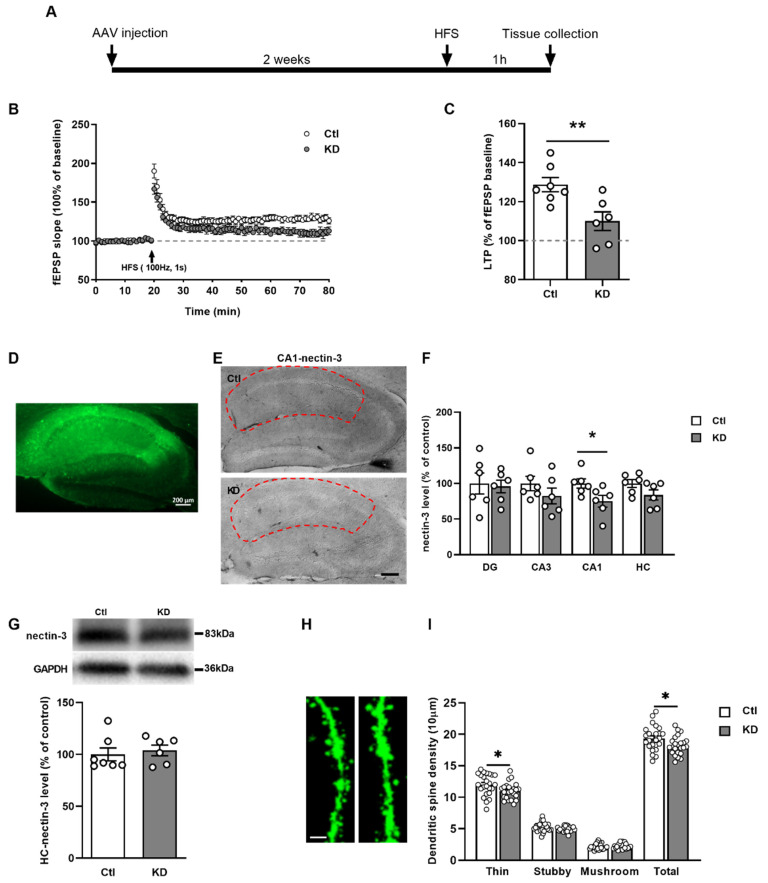
Nectin-3 knockdown in the CA1 region blocked LTP and evoked a reduction in dendritic spines. (**A**) Experimental design. (**B**) Normalized fEPSP of scrambled and knockdown virus-infected brain slices, indicative of attenuated LTP after nectin-3 knockdown (*p* = 0.0089). (**D**) Enhanced GFP image of the virus infection area. Scale bar = 200 µm. (**E**,**F**) Nectin-3 immunoreactivity in the CA1 region (*p* = 0.0399, in red circle), but not in DG, CA3, or the whole hippocampus, was markedly reduced. Scale bars = 200 µm. (**G**) Nectin-3 levels were comparable between control and knockdown groups in the whole hippocampus. (**H**) Representative spines images of both groups. Scale bar = 2 µm. (**I**) Reduced total spines (total: *p* = 0.0253), especially thin spines (*p* = 0.0241) following nectin-3 knockdown in CA1 region. Two-tailed unpaired *t*-test. Data are shown as mean ± SEM. For (**C**,**F**,**G**), dots represented number of animals, and for (**I**), dots represented the number of dendrites. Detailed statistics are provided in Table S1. * *p* < 0.05, ** *p* < 0.01. AAV: adeno-associated virus, ctl: control, DG: dentate gyrus, fEPSP: field excitatory postsynaptic potentials, GAPDH: glyceraldehyde 3-phosphate dehydrogenase, GFP: green fluorescent protein, HC: hippocampus, HFS: high-frequency stimulation, KD: knockdown, LTP: long-term potentiation.

For mimicking the sevo-induced nectin-3 downregulation, we used an AAV-induced knockdown (KD) of nectin-3 to examine the effects of a specific nectin-3 reduction in the CA1 region on LTP and dendritic spine dynamics. Two weeks after the microinjection, the nectin-3 protein levels were significantly reduced in CA1 (Figure 5F—CA1, ctl [100.00 ± 6.60], *n* = 6; KD [75.06 ± 8.25], *t*_10_ = 2.361, *p* = 0.0399, two-tailed unpaired *t*-test), but not in CA3, DG or the whole hippocampus (HC) (Figure 5F–G). Nectin-3 downregulation in CA1 impaired LTP remarkably (Figure 5C, ctl [128.70 ± 3.64%], *n* = 7; KD [110.00 ± 4.77%], *n* = 6; *t*_11_ = 3.171, *p* = 0.0089, two-tailed unpaired *t*-test). Decreased nectin-3 levels in CA1 pyramidal neurons evoked a reduction in total spines (Figure 5I, ctl [19.37 ± 0.40], KD [18.20 ± 0.30], *n* = 2 mice/group, *n* = 24 dendrites/group; *t*_46_ = 2.311, *p* = 0.0253, two-tailed unpaired *t*-test), particularly thin spines (Figure 5I, ctl [11.99 ± 0.35], KD [10.97 ± 0.26], *n* = 2 mice/group, *n* = 24 dendrites/group; *t*_46_ = 2.332, *p* = 0.0241, two-tailed unpaired *t*-test).

### 2.6. Regulation of APP Processing

**Figure 6:**
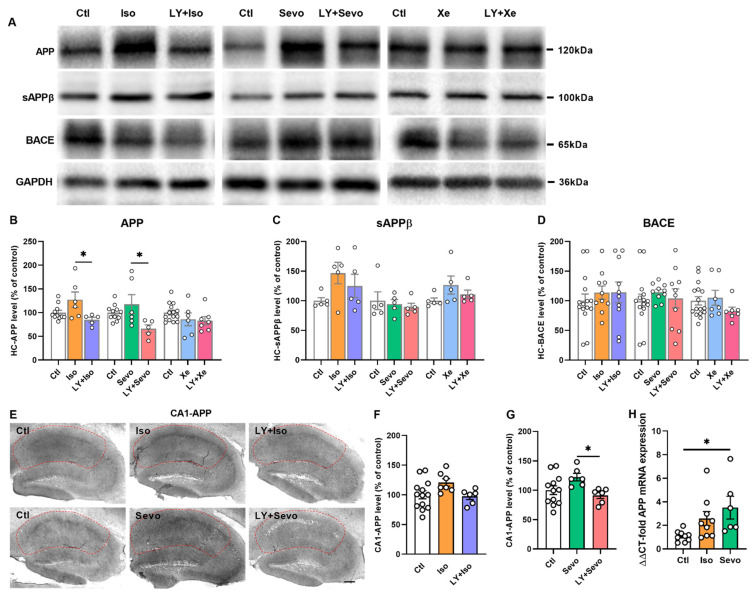
Alteration of hippocampal APP processing-related molecules. (**A**) Representative Western blot images. (**B**–**D**) Normalized protein levels. (**B**) LY + iso and LY + sevo reduced APP levels in comparison with iso or sevo group, respectively (**B**, iso vs. LY + iso: *p* = 0.0238; sevo vs. LY + sevo: *p* = 0.0225). (**C**,**D**) sAPPβ and BACE levels between groups. (**E**–**G**) APP levels in the CA1 region. LY + sevo reduced APP levels compared with the sevo group (**G**, *p* = 0.0421). One-way *ANOVA* followed by *Tukey’s* test. (**H**) Iso trended to enhance hippocampal mRNA levels of APP. Sevo increased mRNA levels of APP in the hippocampus (*p* = 0.0197). Data are shown as mean ± SEM. Dots represent the number of animals. Detailed statistics are provided in the Table S1. * *p* < 0.05. APP: amyloid precursor protein, BACE: β-site APP-cleaving enzyme, ctl: control, GAPDH: glyceraldehyde 3-phosphate dehydrogenase, iso: isoflurane, LY: LY2886721, sAPPβ: soluble ectodomain APP β, sevo: sevoflurane, Xe: xenon.

Next, we measured APP-proteolysis-processing-related protein levels in the hippocampus by Western blot (Figure 6A). Interestingly, the combined application of LY with either iso or sevo downregulated APP expression levels compared with iso/sevo group (Figure 6B, iso [127.40 ± 16.34], *n* = 6; LY + iso [84.95 ± 5.06], *n* = 5; *p* = 0.0238; sevo [117.90 ± 20.09], *n* = 6; LY + sevo [66.15 ± 8.15], *n* = 5; *p* = 0.0225; post hoc with *Tukey’s* test of one-way *ANOVA*). Moreover, sAPPβ and BACE levels in the whole hippocampus were comparable between groups (Figure 6C,E).

Additional immunohistochemistry experiments were performed to detect APP levels in the subregions of the hippocampus. These results showed that, in the CA1 region, APP levels were reduced in LY + sevo groups compared with the sevo group (Figure 6G, sevo [122.20 ± 6.48], *n* = 6; LY + sevo [91.02 ± 5.20], *n* = 6; *p* = 0.0421; post hoc with *Tukey’s* test of one-way *ANOVA*). Incubation with LY, together with either iso or sevo, reduced APP expression levels in CA3 and HC significantly in comparison with iso or sevo treatment alone, respectively (Figure S2A,B; iso vs. LY + iso: CA3—*p* = 0.0061, HC—*p* = 0.0384; sevo vs. LY + sevo: CA3—*p* = 0.0015, HC—*p* = 0.0410; post hoc with *Tukey’s* test of one-way *ANOVA*).

A trend of elevated APP protein levels was observed in the hippocampus (Figure 6B). Likewise, when detecting hippocampal mRNA levels of APP, iso trended to increase the mRNA levels of APP (Figure 6H). Meanwhile, sevo significantly upregulated the mRNA levels of APP in the hippocampus (Figure 6H, ctl [1.09 ± 0.15], *n* = 9; sevo [3.50 ± 0.96], *n* = 6; *p* = 0.0197; *Dunnett’s* test of one-way *ANOVA*).

Western blot and immunohistochemistry procedures were performed to ensure that LY alone did not affect APP levels in the whole or the subregions of the hippocampus (Figure S1E–G).

APP is also a type of cell adhesion molecule supporting the maintenance of dendritic spines and synaptic plasticity [14,27]. Interestingly, we found that APP and nectin-3 were partly colocalized in the hippocampus (Figure S4). We further tested the expression profiles of nectin-3 and APP in the stratum oriens (SO), stratum pyramidale (SP), and stratum lacunosum-moleculare (SLM) layers in the CA1 region (Figure S4E, ctl [0.19 ± 0.01], *n* = 6; LY + sevo [0.15 ± 0.01], *n* = 6; *p* = 0.0104, post hoc with *Tukey’s* test of one-way *ANOVA*).

### 2.7. Iso, Sevo, and Xe Markedly Abolished LTP Respectively

**Figure 7:**
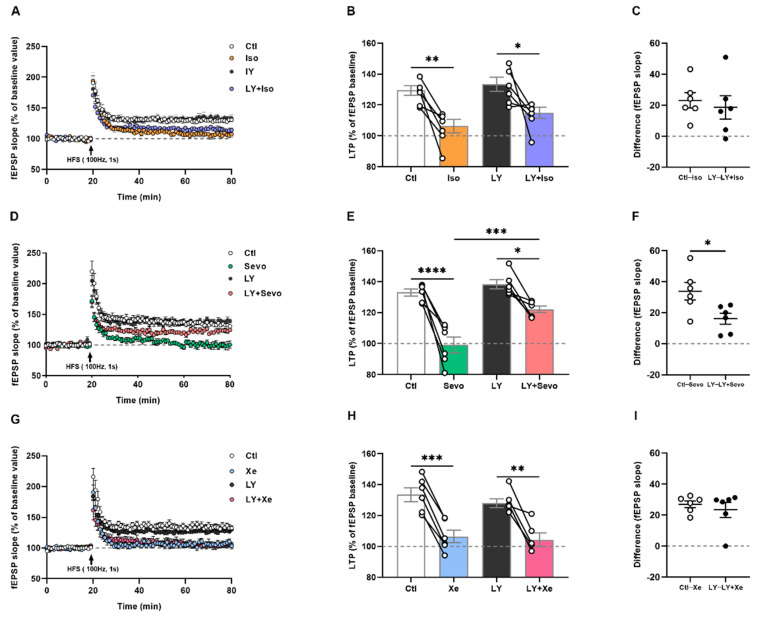
BACE inhibition partly restored sevo-, but not iso- or Xe-induced LTP deficits. Under control and LY application conditions, an HFS train produced stable potentiation but not in the presence of iso, sevo, and Xe. (**A**,**D**,**G**) Normalized fEPSP values under all conditions. Sevo abolished potentiation, which was remarkably reversed by LY exposure (**D**,**E**), ctl vs. sevo: *p* < 0.0001, LY vs. LY + sevo: *p* = 0.0132, sevo vs. LY + sevo: *p* = 0.0005; One-way *ANOVA* followed with *Tukey’s* test) by comparison of the difference between ctl—LY and LY—LY + sevo ((**F**), *p* = 0.0229, two-tailed unpaired *t*-test). In addition, LY application for 2 h in advance was not able to restore the impaired LTP caused by iso or Xe (**A**–**C**,**G**–**I**). Data are presented as mean ± SEM. For box plots in (**B**,**E**,**H**), dots represent the number of animals. Detailed statistics are provided in the Table S1. * *p* < 0.05, ** *p* < 0.01, *** *p* < 0.001, **** *p* < 0.0001. Ctl: control, fEPSP: field excitatory postsynaptic potentials, HFS: high-frequency stimulation, iso: isoflurane, LTP: long-term potentiation, LY: LY2886721, sevo: sevoflurane, Xe: xenon.

Elevated mAβ_1–42_ levels, aberrant spine dynamics and cell adhesions may affect synaptic transmission. Moreover, previous studies have demonstrated a decrease in synaptic transmission as well as LTP in the presence of either iso, sevo, or Xe [2,6,38]. In the next step, we tested how equipotent concentrations of anesthetics affect LTP. By applying 0.6% iso, 1.4% sevo, or 65% Xe for 1.5 h, we observed a marked reduction in fEPSP slopes during the last 10 min of LTP recordings compared to the control (Figure 7B [ctl: 129.40 ± 3.07%] vs. [iso: 106.40 ± 4.31%], *p* = 0.0026; 7e [ctl: 133.00 ± 2.25%] vs. [sevo: 99.18 ± 5.12%], *p* < 0.0001; 7h [ctl: 133.50 ± 4.50%] vs. [Xe: 106.40 ± 4.01%], *p* = 0.0006; *n* = 6 mice/group, post hoc with *Tukey’s* test of one-way *ANOVA*).

### 2.8. Only Sevo-Induced LTP Deficits Were at Least Partly Restored by BACE Inhibition 

Our results demonstrated that BACE inhibition partly reversed the detrimental effects of iso and sevo. Therefore, we further investigated the effects of β-secretase inhibition on attenuated potentiation by either anesthetic. 

To ensure that LY did not affect LTP per se, we found that incubating the slices with LY at 3 µM for 2 h showed no effects on LTP compared with the control conditions (Figure S1C, [133.30 ± 3.31%] vs. [129.70 ± 4.45%], *t*_10_ = 0.6612, *p* = 0.5234, two-tailed unpaired *t*-test, *n* = 6 mice/group). We then incubated slices with the BACE inhibitor LY for 2 h before equipotent concentrations of iso, sevo, or Xe exposure. Analysis of the fEPSP slopes for the last 10 min of LTP revealed that inhibiting β-secretase activity partly restored the negative effects of sevo on LTP, but not that from iso and Xe, as detected by post hoc with *Tukey’s* test of one-way *ANOVA*, respectively (*n* = 6 mice/group): iso [106.40 ± 4.31%] and LY + iso [115.0 ± 3.61%] (Figure 7B, *p* = 0.4282); sevo [99.18 ± 5.12%] and LY + sevo [122.2 ± 2.06%] (Figure 7E, *p* = 0.0005), Xe [106.40 ± 4.01%] and LY + Xe [104.30 ± 4.39%] (Figure 7H, *p* = 0.9823). A two-tailed independent *t*-test (Figure 7F, *t*_10_ = 2.684, *p* = 0.0229, *n* = 6 differences/group) confirmed a significance only between the control—sevo and LY—LY + sevo groups.

The results suggest that equipotent iso, sevo, and Xe blocked LTP, but only sevo-induced LTP deficits were partly reversed by BACE inhibition.

## 3. Discussion

Unraveling the molecular machineries responsible for the effects of inhaled anesthetics on spine dynamics, synaptic connectivity, and interaction with the Aβ-dependent pathophysiology of AD will provide more insight into anesthesia-induced synaptic plasticity.

In this study, we showed that sevo upregulated mAβ_1–42_ levels, downregulated nectin-3 levels, and evoked spine loss. Iso mildly reduced spine density. Although Xe blocked LTP, it did not interfere with spine dynamics and synaptic connectivity. Furthermore, the detrimental effects of sevo were partly restored by BACE inhibition. This may indicate an interference of the inhalational anesthetics with BACE activity and/or synaptic remodeling and synaptic connectivity.

In the hippocampal CA1 region, the pyramidal neurons receive input from CA3 via Schaffer collaterals and synapse to the subiculum in the stratum radiatum layer. Abnormal connectivity of the trisynaptic hippocampal circuit may hamper synaptic connection and transmission [39]. CA1-LTP is an important parameter for measuring synaptic strength. It represents a long-lasting enhancement of synaptic transmission strength following a high-frequency train of stimuli [40].

Dendritic spines can segregate and integrate synaptic signals [41]. Despite the fact that the exact functions of each spine subtype are still not clear, the prevailing opinion is that the thin spines with long necks have a shorter latency and slower decay kinetics of the calcium responses than those in short-necked spines [9,41,42,43].

In the present work, we used the BACE inhibitor LY2886721 to suppress the synthesis of Aβ. LY2886721 is a selective BACE inhibitor without affecting other aspartyl proteases [44]. It has been shown in previous work that LY2886721 decreased Aβ_1-40_ and Aβ_1–42_ deposition in mice, dogs, and healthy human subjects [44,45], which is in line with our finding that LY2886721 reduced mouse Aβ_1–42_.

It has been reported that sevo elevates human Aβ_1–42_ levels [24]. Consistent with the previous reports, sevo upregulated mAβ_1–42_ also in our study. We further showed hereby that mAβ_1–42_ exposure evoked total and thin spine loss and LTP deficits. The upregulation of mAβ_1–42_ could possibly explain our observation that sevo decreased dendritic spine density, especially thin spines, and abolished LTP. Imbalanced APP processing hampers synaptic plasticity [12,46]. We observed increased mRNA levels of APP and a tendency of upregulated APP protein levels in the hippocampus in the presence of sevo. In addition, since BACE is the rate-limiting protease in the Aβ production, the enhanced Aβ synthesis indicates an elevation of BACE activity. The upregulated BACE activity might contribute to sevo-induced synapse loss and LTP deficits. However, further studies are needed in order to measure BACE activity in the presence of higher sevo concentrations reaching at least one minimum alveolar concentration (MAC) [47] for rodents. The cell adhesion molecule nectin-3 is implicated in synaptic plasticity and memory [28,30]. Downregulation of nectin-3 induces hippocampal synapse loss and hippocampus-dependent memory deficits [28,29,30]. In the present study, we found that sevo reduced nectin-3 levels, induced spine loss, and blocked LTP in the CA1 region. Interestingly, mimicking nectin-3 downregulation in CA1 pyramidal neurons by AAV showed similar deficits, given that LTP was attenuated and spine density was also reduced. This suggests that the sevo-induced deficits in CA1-LTP and dendritic spine plasticity could be partially attributed to a reduction in nectin-3 levels. Taken together, the above results indicate that sevo modulates CA1-potentiation by alternating Aβ synthesis, nectin-3 expressions, and spine dynamics in the CA1 region. In addition, the aberrant molecular expression profiles and LTP deficits were partly restored by BACE inhibition, which suggests that sevo may BACE-dependently modulates synaptic dynamics and synaptic transmission.

In line with the previous work, our data confirmed that iso decreases spine density and abolishes CA1-LTP [2,31]. Similar to sevo, iso application also induced a dramatic spine decrease, especially the thin spines in CA1, without upregulation of Aβ production. We assume iso hampered dendritic spine morphology by affecting ion channels, synaptic receptors and triggering potential neuroinflammation and apoptosis [48,49,50,51,52,53,54,55,56]. The observed impairment of CA1-LTP could be correlated with the decreased spine count since they are essential modulators of synaptic activity. Recent studies reported that iso increases mRNA levels of APP, BACE expressions, and Aβ levels [19,20,21,24,25]. However, we found unchanged sAPPβ levels with iso applied at 0.6% (0.21 mM). In addition, in the iso group we observed by means of immunohistochemistry a tendency of an APP increase not only in the CA3 region but also in the whole hippocampus, which was restored by BACE inhibition. Even though these results suggest that iso only mildly modulates the APP proteolysis process, it should be taken into account that iso has been applied at the concentration equivalent to MAC-awake, which is around 0.3 to 0.4 MAC [47,57] (see discussion below).

As a gaseous anesthetic, Xe does not affect dendritic spine dynamics in CA1, although it did block LTP. Previous studies have demonstrated that Xe antagonized LTP, presumably via N-methyl-D-aspartate receptor-dependent mechanisms [6,38]. Compared with iso and sevo, Xe alone, as well as in the presence of LY, maintained APP processing, nectin-3 expressions, and dendritic spine density. This might be indicative of a putative neuroprotective effect of Xe.

Important for the interpretation of the study is the equipotent adjustment of sevo, iso, and Xe concentrations. The rodent MAC for Xe has been shown to be hyperbaric (161 atm [58]). Given that the maximum feasible Xe concentration for slice experiments is limited to 65% [35], we adjusted equipotent concentrations of iso and sevo for comparison, calculated as 0.6% and 1.4%, respectively. Those low concentrations are equivalent to MAC-awake, which has been calculated as approximately a third of MAC for iso and sevo [47,57]. However, it is noticeable that these concentrations, even though they are far below mouse MAC, which is 1.4% for iso and 3.4% for sevo [2], MAC-awake is close to the anesthetic concentration suppressing learning and memory [59,60]. In contrast with some other studies [11,19,20,21,22,23,24,25,26], we applied very low concentrations of sevo and iso at a short application time. On the one hand, this might explain the observed moderate effectiveness of both anesthetics on proteins involved in synaptic connectivity and APP processing-related molecular changes. On the other hand, already subclinical concentrations of iso and sevo clearly produce detrimental effects on synaptic plasticity via Aβ_1–42_-related processes. Intriguingly, the effects on deficits of LTP have been shown to be reversible for both iso and Xe after removal [2,5].

The inhaled anesthetics may affect physiological processes such as autophagy [61], along with alternation of BACE activity. It might partly explain why inhibiting BACE only partially reversed the detrimental effects in the presence of sevo on the molecule level, dendritic spines, as well as LTP. On the other hand, given that BACE has pleiotropic functions, inhibiting that enzyme may hamper hippocampal structure and function in a complex manner [61,62]. For instance, in this study, under the combined application of sevo and the BACE inhibitor, APP and nectin-3 levels in the CA3 region were reduced and accompanied by a reduction in the colocalization profiles of APP and nectin-3 in CA1.

Previous work has shown that nectin-3 is critical for the maintenance of neuronal morphology and function. Downregulation of nectin-3 protein levels in either CA3, DG, or the whole hippocampus evokes a reduction in spines and impairs learning and memory [28,29]. In the present work, we showed that nectin-3 also plays a fundamental role in neuronal structure and function in the CA1 region since a specific knockdown of nectin-3 in the CA1 region blocked LTP and evoked spine loss, especially in thin spines. These observations indicate that nectin-3 is highly involved in spine dynamics and synaptic transmission in the CA1 region of the hippocampus. Interestingly, we found that APP and nectin-3 were colocalized in CA1. Further studies will be necessary in order to gain an understanding of the co-expression and regulatory mechanisms of these two molecules.

In summary, our data indicate that, in the CA1 region, sevo modulates the expression of peptides important for synapse dynamics, neuronal connectivity, and synaptic plasticity, e.g., LTP. BACE is the rate-limiting protease in the Aβ production, so it represents a promising drug target for the treatment of AD. We have shown herein that BACE inhibition was partly able to restore sevo-induced synaptic deficits in the CA1 region. A contribution of sevo in aggravating or even triggering AD is therefore conceivable. The gaseous anesthetic Xe blocked LTP but did not interfere with spine dynamics and synaptic remodeling, indicating a putative neuroprotective mechanism. Our study unraveled the effects of commonly used inhalational anesthetics on the modulation of synaptic plasticity and dendritic spine dynamics and identified BACE as a potential new target for attenuating cognitive deficits as reported after the administration of volatile anesthetics.

## 4. Materials and Methods

### 4.1. Animals and Housing

Adult male C57BL/6J and Thy1-eGFP mice (8–12 weeks old; Charles River, Munich, Germany) were used. All animals were single-housed and were fed under standard conditions (12:12 h light/dark cycle, 22 ± 2 °C, 60% humidity) with free access to tap water and standard mouse food. Mice were randomly assigned to each group. All animal procedures were performed in accordance with a protocol approved by the Technical University Munich and the Government of Upper Bavaria [36,63].

### 4.2. Compounds

The beta-secretase inhibitor LY2886721 (#S2156, Selleck Chemicals, Houston, TX, USA) [45] at a concentration of 3 µM dissolved sequentially in Dimethylsulfoxide (DMSO) and artificial cerebrospinal fluid (aCSF, containing: 125 mM NaCl, 2.5 mM KCl, 25 mM NaHCO3, 2 mM CaCl2, 1 mM MgCl2, 25 mM D-glucose, and 1.25 mM NaH2PO4)) was used.

The mAβ_1–42_ (#32160702, Sigma-Aldrich, Darmstadt, Germany) was firstly suspended in hexafluoroisopropanol (HFIP) at 37 °C for 1.5 h. Then the mixture solution was aliquoted into 5–50 µg portions. Thereafter HFIP was removed by evaporation using a Speedvac for 30 min. When it was completely dry, the aliquots were stored at −20 °C. Before we used it, the lyophilized Aβ_1–42_ was dissolved in DMSO in an ultrasonic water bath. The solution was further dissolved in aCSF to a final concentration of 50 nM.

To assure a sufficient oxygen supply and a stable pH level of 7.2–7.4 during Xe application, the maximum Xe concentration used in the slice experiments was limited to 65% (1.9 mM in aCSF) [35]. The Xe application was thus conducted by gassing a Xe gas mixture (65% Xe, 30% O_2_, 5% CO_2_) [6,36,41] and a nitrogen mixture gas (65% N_2_, 30% O_2_, 5% CO_2_) as a control, both co-applied in exchange with carbogen gas (95% O_2_/5% CO_2_) to the aCSF. For the sake of comparability and ensuring equipotent MAC-awake [48,59] concentrations of anesthetics, we adjusted the concentrations of iso to 0.6% (0.21 mM) and sevo to 1.4% (0.29 mM), which were added via a vapor to the carbogen gas.

### 4.3. Slices Preparation

Mice were anesthetized with iso and decapitated. The brains were removed and quickly placed in ice-cold aCSF (Sagittal slices, including the hippocampus, were prepared at a thickness of 350 μm using a vibratome (VT1000 S, Leica, Germany). The slices were incubated at 35 °C for 0.5 h, then allowed to recover at room temperature (RT) for at least 1 h. A sufficient mixture of 95% O_2_/5% CO_2_ (carbogen gas) was applied, which led to a pH level of 7.4 during the preparation, incubation, and recording stages [6,35].

### 4.4. Field Excitatory Postsynaptic Potentials (fEPSPs)

The fEPSPs were recorded as previously described [40,64]. Recordings were taken in the hippocampal CA1 stratum radiatum region with two bipolar tungsten electrodes (insulated to the tip; 50 µm in diameter). The electrodes were placed at either side of the recording pipette (glass borosilicate micropipette, Clark Electromedical Instruments, Pangbourne Reading, United Kingdom) and filled with aCSF, resulting in an open tip resistance of 1–2 MΩ. Non-overlapping populations of fibers of the Schaffer collateral-associated commissural pathway were stimulated with two electrodes, one for internal control and another for treatment. The fEPSPs were evoked alternatively by delivering a test stimulus (50 µs) via one of two electrodes at a stimulus interval of 15 s. Two average consecutive fEPSPs were recorded for noise minimization. The stimulation was adjusted to achieve a response of approximately 50% of the maximal obtainable response for the baseline recordings. The synaptic transmission strength was measured with the slope of the rising phase (20–80% peak amplitude) of the fEPSPs. To obtain a baseline recording, fEPSP slopes were recorded for at least 20 min after they stabilized. For LTP induction, a high-frequency stimulation (HFS) train of 100 pulses at 100 Hz was applied to the Schaffer collateral pathway with an additional 1 h fEPSP slopes recordings. The stimulation rate was unchanged during the recording. Data were monitored using WinLTP, version 1.11b (Win LTP Ltd., Centre for Synaptic Plasticity, School of Physiology & Pharmacology, University of Bristol, Bristol, UK). Slopes were normalized with the responses recorded within the final 20 min before HFS.

The anesthetics or LY2886721 were applied after HFS1 was triggered. Thereafter, the first LTP was maintained for 1 h; either iso, sevo, or Xewas applied. After 1.5 h, HFS2 was delivered, and the fEPSPs were recorded for an additional 1 h. The fEPSP slopes recorded 20 min before HFS2 were used as the baseline data for the secondary LTP.

For the LY2886721 + anesthetics application experiments, LY2886721 was applied and mixed well in the aCSF before the slices were transferred to the recording chamber. After 2 h of incubation, HFS1 was triggered, and fEPSPs were monitored for 1 h. Either iso, sevo, or Xewas applied for 1.5 h before the trigger of HFS2.

For the experiments conducted with slices from AAV-treated animals, two electrodes were used for the fEPSP slopes recordings, but LTP was induced in one input only.

### 4.5. Stereotaxic Virus Microinjection

We used AAV9 vectors to suppress nectin-3 expression levels in the CA1 region. Previous work has validated the short hairpin RNA (shRNA) sequence targeting nectin-3 5′-TGTGTCCTGGAGGCGGCAAAGCACAACTT-3′ [28,29,30]. AAV-shNectin-3 (AAV9-CMV-Nectin3.shRNA-terminator-GFP-hGH-amp, >1 × 10^9^ viral genomes/mL) and scrambled control virus (AAV9-CMV-Scrambled.shRNA-terminator-GFP-hGH-amp, 3.5 × 10^12^ viral genomes/mL) were presented by Applied Biological Materials Inc. (abm, New York, NY, USA).

Stereotaxic microinjection was performed as previously described [29]. Briefly, 0.5 μL virus was delivered bilaterally to the CA1 region using a 1 μL Hamilton syringe (Hamilton, 7001) with a 33-gauge needle (Hamilton, 65461-01). The injected site was located as follows: 1.8 mm posterior to bregma, 1.2 mm lateral from midline, and 1.4 mm dorsoventral from the dura. The virus was applied by a pump at the rate of 0.05 μL/min. The needle was left at the site for an additional 2 min once the injection was performed. To allow sufficient virus infection, mice were allowed to recover for two weeks prior to the LTP experiment.

### 4.6. Immunostaining and Image Analysis

Right after the extracellular recordings, slices were collected, fixed with 4% paraformaldehyde for 2 days, cryoprotected with 30% sucrose for 3 days, and further cut into 40 µm thickness sections using cryostat (CryoStar NX70, Thermo Fisher Scientific, Bremen, Germany) for further staining.

For immunohistochemistry experiments, free-floating sections were simultaneously treated with 0.1 M PBS (3 × 10 min), hydrogen peroxide block (10 min), 0.1 M PBS (3 × 10 min), 0.1% normal goat serum (1 h) at RT, and were then labeled with mouse anti-APP (22C11) (1:1000; #14-9749-82, Thermo Fisher Scientific, Bremen, Germany) or mouse anti-nectin-3 (H-11) (1:1000; sc-271611, Santa Cruz, CA, USA) at 4 °C overnight. On the second day, sections were washed with 0.1 M PBS (3 × 10 min), incubated with biotinylated goat anti-mouse polyvalent (RT, 2 h), washed with 0.1 M PBS (3 × 10 min), and treated with streptavidin peroxidase (RT, 2 h). After rinsing, sections were stained with 3,3′-Diaminobenzidine Horseradish Peroxidase Color Development Kit DAB (Chromogen and DAB Substrate (1:50)) for 10 min. Thereafter, sections were washed, transferred onto slides, dehydrated with EtOH (70%, 95%, and 100%, 5 min of each) and Xylene (3 × 5 min) sequentially, and coverslipped. Images from 4 to 8 sections per animal were acquired with the Zeiss Microscope (Carl Zeiss Microscopy GmbH, Munich, Germany) to quantity the immunoreactivity of APP and nectin-3 levels. As previously described [29], we determined the relative protein levels by differentiating optical density values analyzed by ImageJ (National Institute of Health, Bethesda, MD, USA) between the region of interest and the corpus callosum, which lacked staining and was measured as the background. The results were shown as a percentage of the control group in each region.

For immunofluorescence experiments, free-floating sections were simultaneously treated with 0.1 M PBS (3 × 10 min) and 0.1% normal goat serum (1 h) at RT and were thereafter labeled with mouse anti-APP (22C11) (1:1000; #14-9749-82, Thermo Fisher Scientific, Bremen, Germany) or rabbit anti-nectin-3 (1:1000; sc-ab63931, abcam, Cambridge, UK) or rabbit anti-GFP (1:500; #A-11122, Thermo Fisher Scientific, Bremen, Germany) at 4 °C overnight. On the second day, sections were rinsed with 0.1 M PBS (3 × 10 min) and then labeled with Alexa Fluor 488/594-conjugated goat anti-mouse/rabbit secondary antibody (1:500; Invitrogen, Carlsbad, CA, USA) for 2 h at room temperature. Afterward, sections were washed, transferred onto slides, and coverslipped. For the evaluation of the colocalization patterns of APP and nectin-3, images from 4 to 8 sections per animal were acquired with the Zeiss Microscope (Carl Zeiss Microscopy GmbH, Munich, Germany) and analyzed with Imaris software (Oxford instruments, Oxfordshire, UK).

### 4.7. ELISA

Mouse Aβ_1–42_ levels were detected by a sandwich ELISA assay (KBM3441, Invitrogen, Berlin, Germany). A sufficient amount of hippocampal tissues was collected and weighted. Tissues were homogenized with guanidine buffer (8× the tissue mass) at RT for 4 h, diluted four-fold with cold 0.1 M PBS including 1× protease inhibitor cocktail, and centrifuged at 12,000× for 30 min at 4 °C. The supernatant was collected and added to the appropriate wells at RT for 4 h. After washing, samples were sequentially treated with mAβ_1–42_ detection antibody (RT, 1 h), wash buffer (4 times), and anti-rabbit IgG HRP (RT, 30 min). After rinsing, stabilized chromogen was added and incubated for 30 min at RT in the dark. Stop solution was applied, and the absorbance at 450 nm was read by an absorbance microplate reader. The results were described as a percentage of the control group.

### 4.8. Western Blot

Hippocampus was dissected and frozen after LTP experiments. Tissues were homogenized in ice-cold lysis buffer (2% 50× Protease Inhibitor Cocktail tables (Roche, Switzerland)), 0.1% Pepstatin (P5318, Roche, Switzerland), 0.1% 100× phenylmethylsulfonyl fluoride (#7626, Sigma Aldrich, Darmstadt, Germany) and 97% PIPA buffer (R0278, Sigma Aldrich, Darmstadt, Germany). Thereafter, samples were centrifuged at 12000 RPM for 30 min at 4 °C. The supernatant was collected, and the protein concentrations were determined by a pre-diluted protein standard assay. Samples containing 20 μg of protein were separated by sodium dodecyl sulfate-polyacrylamide gels (10%) and transferred onto polyvinylidene difluoride membranes (Amersham Hybond low fluorescence membrane; GE Healthcare, Munich, Germany). After rinsing with TBST and blocking with 10% Roti-Block (Roth, Karlsruhe, Germany) at RT for 1 h, membranes were labeled with primary antibodies (mouse anti-APP (22C11), 1:2000, #14-9749-82, Thermo Fisher; rabbit anti-BACE, 1:2000, #5606S, Cell Signaling; rabbit anti-GAPDH, 1:5000, PA1-987, Thermo Fisher Scientific; rabbit anti-nectin-1, 1:2000, ab-66985, abcam; mouse anti-nectin-3 (H-11), 1:1000; sc-271611, Santa Cruz; rabbit anti-sAPPβ, 1:2000, #813401, Biolegend) overnight at 4 °C. Following incubation with horseradish peroxidase-conjugated secondary antibodies (1:10,000, anti-rabbit or mouse IgG; Thermo Fisher Scientific, Bremen, Germany) for 1 h at RT, bands were visualized using an Enhanced Chemiluminescence detection kit and BioRad Image Lab. The results were normalized as a percentage of the control group. Each assay was repeated 2–4 times.

### 4.9. Quantitative Real-Time PCR (RT-PCR)

RNA was extracted from the hippocampus using an RNA extraction kit (RNeasy kit Qiagen, Germany). RNA (0.5–1 µg) was reversely transcribed using QuantiTect Rev. Transcription Kit (Qiagen). RT-PCR analysis was conducted using QuantiNova SYBR green PCR kit (Qiagen) on a Rotor GeneQ (Qiagen). The APP primer sets contained 10 pM of each sense primer 5′-GACCCGTCAGGGACCAAAAC-3′ and the antisense primer 5′-AACGGTAAGGAATCACGATGTG-3′ (Metabion, Bayern, Germany). The controlled 18S rRNA primer sets were as follows: sense 5′-GTAACCCGTTGAACCCCATT-3′, antisense 5′-CCATCCAATCGGTAGTAGCG-3′ (Metabion, Bayern, Germany). The cycle threshold (Ct) values of the APP genes were normalized to the CT values of the 18S rRNA gene for the quantification of the relative APP mRNA expressions. The results were shown as fold change (=2^−ΔΔCt^). The relative mRNA expressions of APP gene were calculated using the following equations: ΔCt = Ct (APP) − Ct (18S rRNA), ΔΔCt = ΔCt (iso/sevo group) − ΔCt (control group).

### 4.10. Classification of Dendritic Spines

Thy1-eGFP mice were used for the dendritic spine analysis. After the experimental procedure, slices were collected, fixed with 4% PFA for 2 days, and cryoprotected with 30% sucrose for 3 days. Slices at a 40 µm thickness were prepared using a cryostat (CryoStar NX70, Thermo Fisher Scientific, Bremen, Germany). After washing with PBS, the slices were transferred onto slides and coverslipped.

Images containing apical oblique dendritic spines in the CA1 region were obtained using a confocal microscope (Leica SP8, Wetzlar, Germany) at 0.3 µm interval z-stacks with a 60× oil-immersion objective. Dendritic segments (20–80 µm in length, 6–8 dendrites from 6 to 8 pyramidal neurons per mouse) from the stratum radiatum in the CA1 region were analyzed with Leica Application Suite X software (Leica, Wetzlar, Germany). Dendritic spines were categorized as thin, mushroom, and stubby subtypes, based on established criteria as follows [29,30]: (1) thin spines—long and thin protrusions with a bulbous head; (2) mushroom spines—protrusions with a small neck and a large head; and (3) stubby spines—protrusions closely connect to the dendritic shaft and lack a clear neck. The spine density was presented as the number of spines per 10 μm of the dendrite.

### 4.11. Statistical Analysis

Statistics were performed using GraphPad Prism 8 software. For the comparisons of fEPSP slopes, mAβ_1–42_ levels, hippocampal protein levels, and dendritic spine density between three or four groups, one-way analysis of variance one-way (*ANOVA*) followed by *Tukey’s* post-hoc multiple comparisons test were used. A two-tailed unpaired *t*-test (homogeneity of variance was met) or a two-tailed *Mann–Whitney* test (homogeneity of variance was not met) was performed for the comparisons of the two groups. Data were reported as mean ± standard error of the mean (SEM). The statistical significance was defined at *p* < 0.05.

## Data Availability

Not applicable.

## Supplementary Materials

The following supporting information can be downloaded at: https://www.mdpi.com/article/10.3390/ijms23126637/s1.
