# Supplementary material for: Beta-Site Amyloid Precursor Protein-Cleaving Enzyme Inhibition Partly Restores Sevoflurane-Induced Deficits on Synaptic Plasticity and Spine Loss
Source: Int J Mol Sci. 2022 Jun 14;23(12):6637. doi: 10.3390/ijms23126637 (PMC9223703; doi:10.3390/ijms23126637)
Supplement: Supplementary file 1 [file ijms-23-06637-s001.zip › ijms-1713082-supplementary.pdf]

Supplementary Materials.

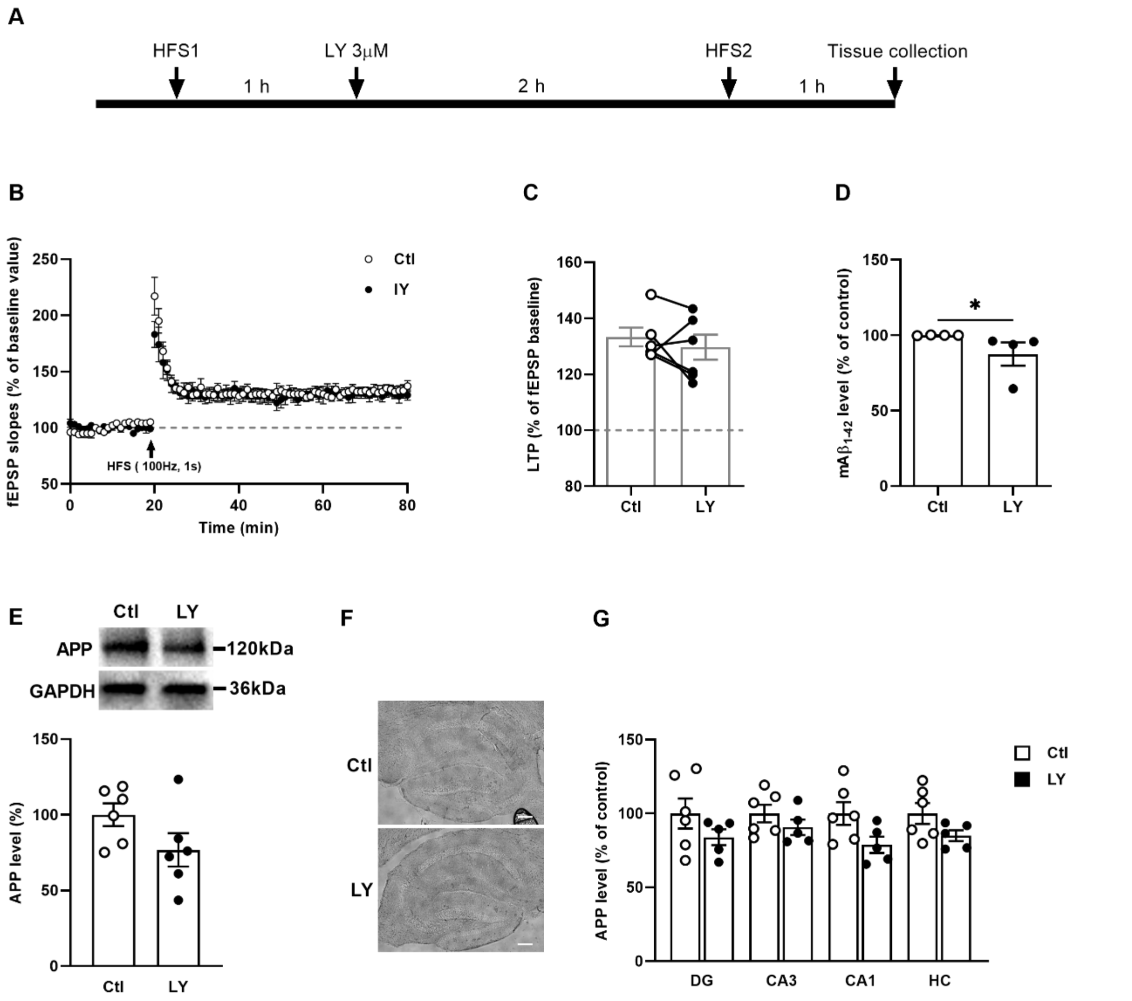

Figure S1. Partial reduction of amyloid beta production by beta-secretase inhibitor LY2886721 did not block LTP. (A) Experimental design. (B) Normalized fEPSP values under control and LY conditions. (C) LY of 3 μM for 2 h treatment did not block LTP. (D) Mouse $A\beta_{1-42}$ levels in the hippocampus was significantly reduced after LY exposure (p = 0.0286, two-tailed *Mann-Whitney* test). LY did not change APP levels in the whole (E) or the subregions of hippocampus (F,G). Scale bars = 200 μm. Data are shown as mean ± SEM. Dots in C-E and G represent the number of animals. Detailed statistics are provided in Table S1. $^*p < 0.05$. APP: amyloid precursor protein, Aβ: amyloid beta, ctl: control, DG: dentate gyrus, fEPSP: field excitatory postsynaptic potentials, GAPDH: Glyceraldehyde 3-phosphate dehydrogenase, HC: hippocampus, HFS: high-frequency stimulation, iso: isoflurane, LTP: long-term potentiation, LY: LY2886721, sevo: sevoflurane, Xe: Xenon.

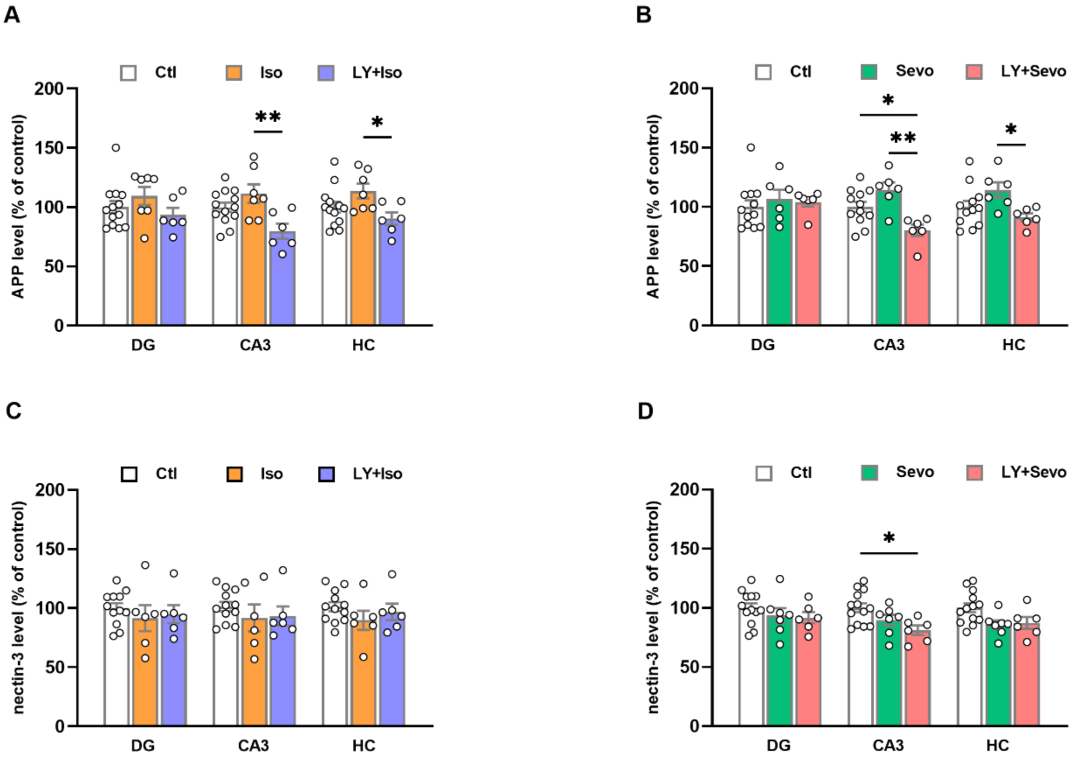

Figure S2. APP and nectin-3 levels in DG, CA3 and HC regions. (A) LY+iso reduced APP levels either in CA3 ($p = 0.0061$) or the whole hippocampus ($p = 0.0384$) compared with iso group. (B) APP levels in LY+sevo were decreased in the CA3 region compared with the control group (p = *0.0322*). Moreover, in comparison with sevo group, APP levels were reduced either in CA3 (p = *0.0015)* or the whole hippocampus (p = *0.0410*). (C) No alternations of nectin-3 protein levels under any conditions. (D) LY+sevo downregulated nectin-3 expression levels in the CA3 region in comparison with the control group (p = *0.0153*). One-way *ANOVA* followed with *Tukey's* test. Data are shown as mean ± SEM. Dots represent the number of animals. Detailed statistics are provided in the Table S1. *$p < 0.05$, **$p < 0.01$. APP: amyloid precursor protein, ctl: control, DG; dentate gyrus, HC: hippocampus, iso: isoflurane, LY: LY2886721, sevo: sevoflurane.

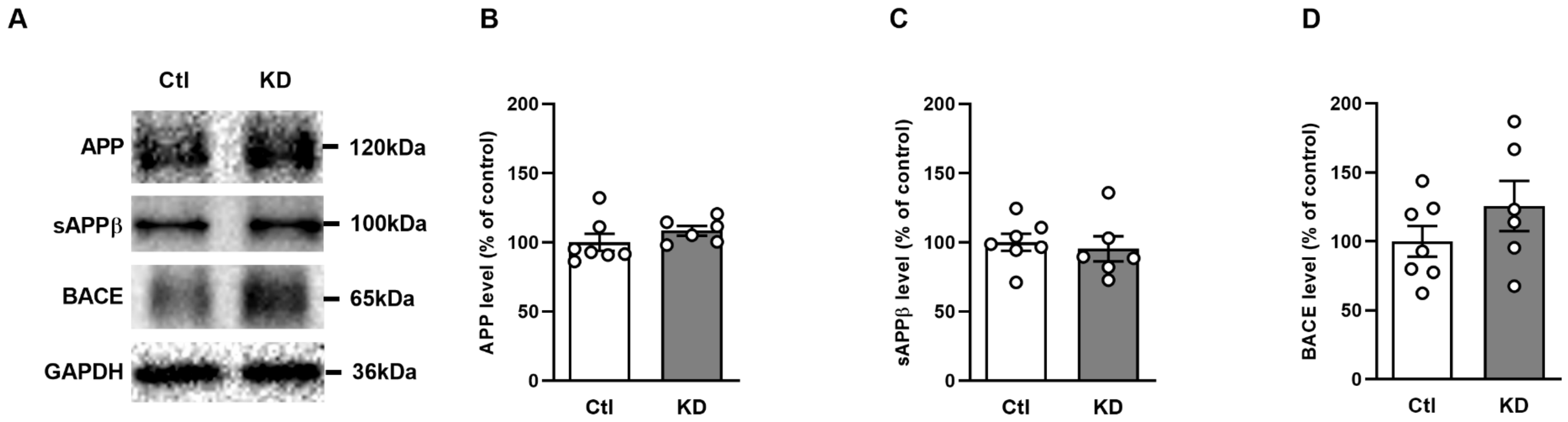

Figure S3. Knockdown of nectin-3 in CA1 region did not significantly change hippocampal APP, sAPPβ, or BACE levels. (A) Representative images of western blot of all groups. (B–D) Normalized hippocampal protein levels. Data are shown as mean ± SEM. Dots in B-D and G represent the number of animals. Detailed statistics are provided in Table S1. APP: amyloid precursor protein, BACE: β-site APP-cleaving enzyme, ctl: control, GAPDH: Glyceraldehyde 3-phosphate dehydrogenase, KD: knockdown, sAPPβ: soluble ectodomain amyloid precursor protein β.

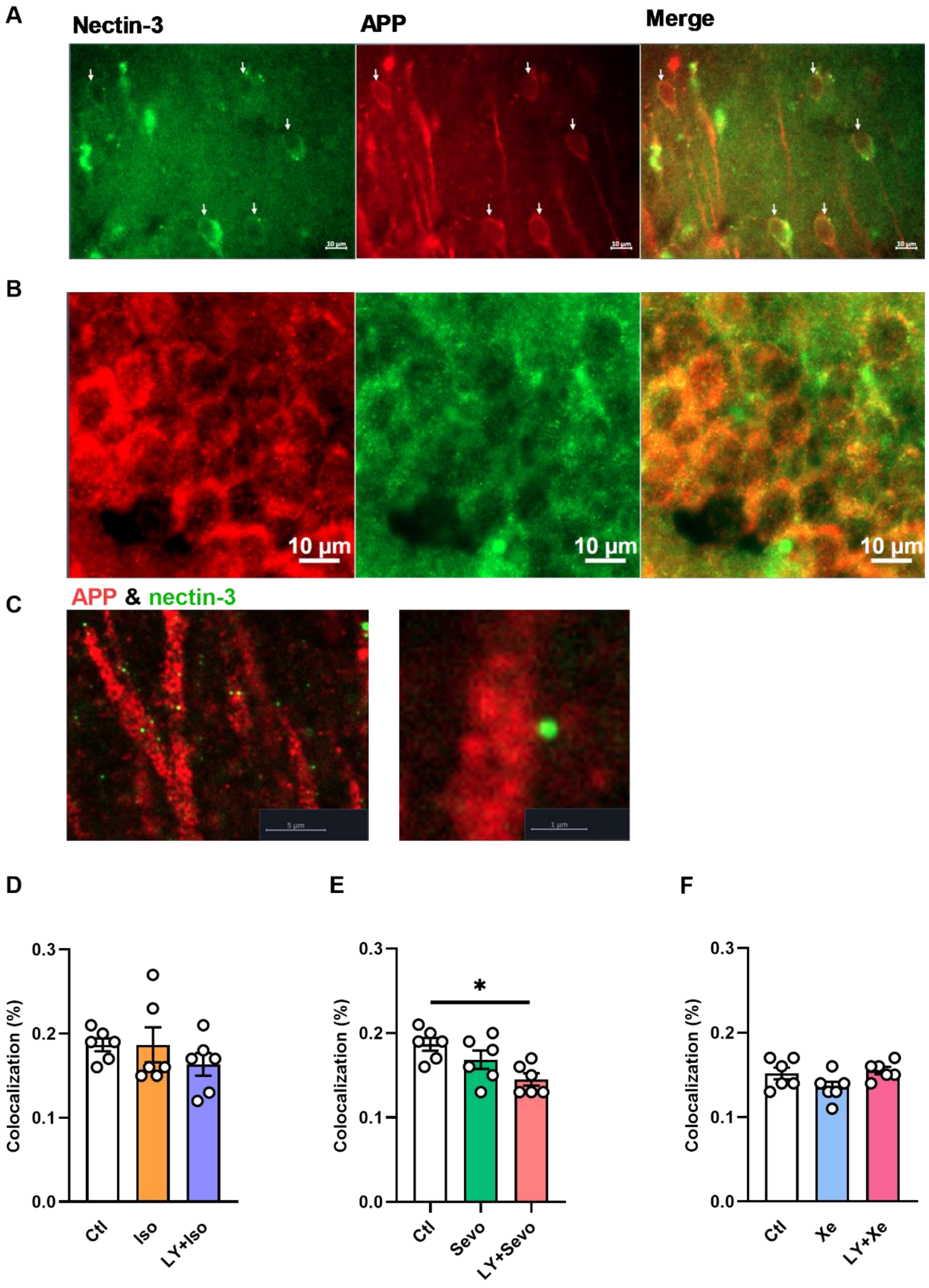
A
Nectin-3
APP
Merge
B
10 µm
10 µm
10 µm
C
APP & nectin-3
D
0.3
0.2
0.1
0.0
Colocalization (%)
Ctl
Iso
LY+Iso
E
*
Ctl
Sevo
LY+Sevo
F
Ctl
Xe
LY+Xe

Figure S4. Double staining of APP and nectin-3 in the CA1 region. Co-staining profiles of APP and nectin-3 in the SO (A), SP (B) and SLM (C) layers in CA1 region. (D–F) Colocalization percentage of APP and nectin-3 in CA1 region. LY+Sevo reduced colocalizational percent compared with control group (E, $p = 0.0104$). One-way *ANOVA* followed with *Tukey's* test. Scale bars = 10 or 1 μm. Data are shown as mean ± SEM. Dots represent the number of animals. Detailed statistics are provided in the Table S1. $^*p < 0.05$. APP: amyloid precursor protein, ctl: control, iso: isoflurane, LY: LY2886721, sevo: sevoflurane, SLM: Stratum lacunosum-molecular layer, SO: Stratum oriens, SP: Stratum pyramidal, Xe: Xenon.

Table S1. Statistical results.

Figure	*Statistical method*	*Factors*	*Statistical results*	*Post hoc method*	*Post hoc results*	*Sample size*
1a	One-way ANOVA	condition	F (2, 15) = 0.4609, P = 0.6394			6 mice/group
1b	One-way ANOVA	condition	F (2, 18) = 8.397, P = 0.0027	Tukey's test	Ctl vs. Sevo: P = 0.0134<br>Ctl vs. LY+Sevo: P = 0.6633<br>Sevo vs. LY+Sevo: P = 0.0034	Ctl: n = 8 mice<br>Sevo: n = 7 mice<br>LY+Sevo: n = 6 mice
1c	One-way ANOVA	condition	F (2, 15) = 0.4686, P = 0.6347			6 mice/group
2f	Two-tailed unpaired t test	condition	t_{10} = 3.297, P = 0.0081			6 mice/group
2h	Two-tailed unpaired t test	condition	Thin: t_{10} = 5.323, P = 0.0003;<br>Stubby: t_{10} = 1.592, P = 0.1424;<br>Mushroom: t_{10} = 1.610, P = 0.1385;<br>Total: t_{10} = 6.078, P = 0.0001			6 mice/group
3d–1	One-way ANOVA	condition	F (2, 13) = 16.16, P = 0.0003	Tukey's test	Ctl vs. Iso: P = 0.0006<br>Ctl vs. LY+Iso: P = 0.0036<br>Iso vs. LY+Iso: P = 0.6464	Ctl: n = 8 mice<br>Iso: n = 4 mice<br>LY+Iso: n = 4 mice

3d–2	One-way ANOVA	condition	F (2, 15) = 6.855, P = 0.0077	Tukey’s test	Ctl vs. Sevo: P = 0.0066<br>Ctl vs. LY+Sevo: P = 0.1351<br>Sevo vs. LY+Sevo: P = 0.2265	Ctl: n = 8 mice<br>Sevo: n = 4 mice<br>LY+ Sevo: n = 6 mice
3d–3	One-way ANOVA	condition	F (2, 8) = 0.7048, P = 0.5225			Ctl: n = 4 mice<br>Xe: n = 3 mice<br>LY+Xe: n = 4 mice
3e–1	One-way ANOVA	condition	F (2, 13) = 1.872, P = 0.1931			Ctl: n = 8 mice<br>Iso: n = 4 mice<br>LY+Iso: n = 4 mice
3e–2	One-way ANOVA	condition	F (2, 15) = 0.8358, P = 0.4528			Ctl: n = 8 mice<br>Sevo: n = 4 mice<br>LY+ Sevo: n = 6 mice
3e–3	One-way ANOVA	condition	F (2, 8) = 1.007, P = 0.4075			Ctl: n = 4 mice<br>Xe: n = 3 mice<br>LY+Xe: n = 4 mice
3f–1	One-way ANOVA	condition	F (2, 13) = 3.513, P = 0.0603			Ctl: n = 8 mice<br>Iso: n = 4 mice<br>LY+Iso: n = 4 mice
3f–2	One-way ANOVA	condition	F (2, 15) = 0.4234, P = 0.6624			Ctl: n = 8 mice<br>Sevo: n = 4 mice<br>LY+ Sevo: n = 6 mice
3f–3	One-way ANOVA	condition	F (2, 8) = 2.370, P = 0.1555			Ctl: n = 4 mice<br>Xe: n = 3 mice<br>LY+Xe: n = 4 mice
3g–1	One-way ANOVA	condition	F (2, 13) = 13.04, P = 0.0008	Tukey’s test	Ctl vs. Iso: P = 0.0012<br>Ctl vs. LY+Iso: P = 0.0095<br>Iso vs. LY+Iso: P = 0.6104	Ctl: n = 8 mice<br>Iso: n = 4 mice<br>LY+Iso: n = 4 mice

3g–2	One-way ANOVA	condition	F (2, 15) = 3.803, P = 0.0461	Tukey's test	Ctl vs. Sevo: P = 0.0370<br>Ctl vs. LY+Sevo: P = 0.6194<br>Sevo vs. LY+Sevo: P = 0.1964	Ctl: n = 8 mice<br>Sevo: n = 4 mice<br>LY+ Sevo: n = 6 mice
3g–3	One-way ANOVA	condition	F (2, 8) = 0.1117, P = 0.8957			Ctl: n = 4 mice<br>Xe: n = 3 mice<br>LY+Xe: n = 4 mice
4c–1	One-way ANOVA	condition	F (2, 21) = 1.551, P = 0.2353			Ctl: n = 12 mice<br>Iso: n = 7 mice<br>LY+Iso: n = 5 mice
4c–2	One-way ANOVA	condition	F (2, 23) = 1.631, P = 0.2196			Ctl: n = 12 mice<br>Sevo: n = 7 mice<br>LY+ Sevo: n = 5 mice
4c–3	One-way ANOVA	condition	F (2, 23) = 0.1350, P = 0.8744			Ctl: n = 14 mice<br>Xe: n = 5 mice<br>LY+Xe: n = 7 mice
4d	One-way ANOVA	condition	F (2, 21) = 1.942, P = 0.1684			Ctl: n = 12 mice<br>Iso: n = 6 mice<br>LY+Iso: n = 6 mice
4e	One-way ANOVA	condition	F (2, 23) = 4.302, P = 0.0259	Tukey's test	Ctl vs. Sevo: P = 0.0205<br>Ctl vs. LY+Sevo: P = 0.4258<br>Sevo vs. LY+Sevo: P = 0.3932	Ctl: n = 13 mice<br>Sevo: n = 7 mice<br>LY+ Sevo: n = 6 mice
5c	Two-tailed unpaired t test	condition	t_{11} = 3.171, P = 0.0089			Ctl: n = 7 mice;<br>KD: n = 6 mice
5f	Two-tailed unpaired t test	condition	DG: t_{10} = 0.2415, P = 0.8141;			n = 6 mice/group

			CA3: t_{10} = 1.169, P = 0.2695<br>CA1: t_{10} = 2.361, P = 0.0399<br>HC: t_{10} = 1.770, P = 0.1072			
5g	Two-tailed t test	condition	t_{11} = 0.4507, P = 0.6609			Ctl: n = 7 mice; KD: n = 6 mice
5i–Thin	Two-tailed t test	condition	t_{46} = 2.332, P = 0.0241			Ctl: n = 2 mice, n = 12 dedrites;<br>KD: n = 2 mice, n = 12 dedrites
5i–Stubby	Two-tailed t test	condition	t_{46} = 1.861, P = 0.0691			
5i–Mushroom	Two-tailed Mann-Whitney test	condition	U = 276.5, P = 0.8184			
5i–Total	Two-tailed t test	condition	t_{46} =2.311, P = 0.0253			
6b–1	One-way ANOVA	condition	F (2, 19) = 4.511, P = 0.0249	Tukey's test	Ctl vs. Iso: P = 0.0913;<br>Ctl vs. LY+Iso: P = 0.4932;<br>Iso vs. LY+Iso: P = 0.0238;	Ctl: n = 11 mice<br>Iso: n = 6 mice<br>LY+Iso: n = 5 mice
6b–2	One-way ANOVA	condition	F (2, 19) = 4.386, P = 0.0272	Tukey's test	Ctl vs. Sevo: P = 0.4638;<br>Ctl vs. LY+Sevo: P = 0.1062;<br>Sevo vs. LY+Sevo: P = 0.0225	Ctl: n = 11 mice<br>Sevo: n = 6 mice<br>LY+ Sevo: n = 5 mice
6b–3	One-way ANOVA	condition	F (2, 24) = 1.521, P = 0.2388			Ctl: n = 14 mice<br>Xe: n = 6 mice<br>LY+Xe: n = 7 mice

6c–1	One-way ANOVA	condition	F (2, 12) = 2.211, P = 0.1522			5 mice/group
6c–2	One-way ANOVA	condition	F (2, 12) = 0.2506, P = 0.7823			5 mice/group
6c–3	One-way ANOVA	condition	F (2, 12) = 1.745, P = 0.2162			5 mice/group
6d–1	One-way ANOVA	condition	F (2, 32) = 0.4113, P = 0.6663			Ctl: n = 15 mice<br>Iso: n = 10 mice<br>LY+Iso: n = 10 mice
6d–2	One-way ANOVA	condition	F (2, 32) = 0.4165, P = 0.6629			Ctl: n = 15 mice<br>Sevo: n = 5 mice<br>LY+ Sevo: n = 5 mice
6d–3	One-way ANOVA	condition	F (2, 27) = 1.307, P = 0.2872			Ctl: n = 16 mice<br>Xe: n = 7 mice<br>LY+Xe: n = 7 mice
6f	One-way ANOVA	condition	F (2, 32) = 2.987, P = 0.0703			Ctl: n = 13 mice<br>Iso: n = 7 mice<br>LY+Iso: n = 6 mice
6g	One-way ANOVA	condition	F (2, 21) = 3.695, P = 0.0422	Tukey's test	Ctl vs. Sevo: P = 0.1055;<br>Ctl vs. LY+Sevo: P = 0.6678;<br>Sevo vs. LY+Sevo: P = 0.0421	Ctl: n = 12 mice<br>Sevo: n = 6 mice<br>LY+ Sevo: n = 6 mice
6h	One-way ANOVA	condition	F (2, 21) = 3.695, P = 0.0422	Dunnett's test	Ctl vs. Iso: P = 0.1199;<br>Ctl vs. Sevo: P = 0.0197	Ctl: n = 9 mice<br>Iso: n = 9 mice<br>Sevo: n = 6 mice
7b	One-way ANOVA	condition	F (3, 20) = 10.31, P = 0.0003	Tukey's test	Ctl vs. Iso: P = 0.0026;<br>Ctl vs. LY: P = 0.8841;<br>Ctl vs. LY+iso: P = 0.0742;	6 mice/group

					Iso vs. LY: P = 0.0005;<br>Iso vs. LY+iso: P = 0.4282;<br>LY vs. LY+iso: P = 0.0161	
7c	Two-tailed unpaired t test	condition	$t_{10} = 0.5043$,<br>P = 0.6250			6 differences/group
7e	One-way ANOVA	condition	F (3, 20) = 26.98,<br>P < 0.0001	Tukey's test	Ctl vs. Sevo: P < 0.0001;<br>Ctl vs. LY: P = 0.6793;<br>Ctl vs. LY+Sevo: P = 0.1322;<br>Sevo vs. LY: P < 0.0001;<br>Sevo vs. LY+Sevo: P = 0.0005;<br>LY vs. LY+Sevo: P = 0.0132	6 mice/group
7f	Two-tailed unpaired t test	condition	$t_{10} = 2,684$,<br>P = 0.0229			6 differences/group
7h	One-way ANOVA	condition	F (3, 20) = 13.87,<br>p < 0.0001	Tukey's test	Ctl vs. Xe: P = 0.0006;<br>Ctl vs. LY: P = 0.7678;<br>Ctl vs. LY+Xe: P = 0.0003;<br>Xe vs. LY: P = 0.0053;<br>Xe vs. LY+Xe: P = 0.9823<br>LY vs. LY+Xe: P = 0.0023;	6 mice/group
7i	Two-tailed Mann-Whitney test	condition	U = 16,<br>P = 0.8182			6 differences/group
S1. c	Two-tailed unpaired t test	condition	$t_{10} = 0.6612$,<br>P = 0.5234			6 mice/group

S1. d	Two-tailed Mann-Whitney test	condition	U = 0,<br>P = 0.0286			4 mice/group
S1. e	Two-tailed unpaired t test	condition	t_{10} = 1.752,<br>P = 0.1103			6 mice/group
S1. g–DG	Two-tailed unpaired t test	condition	t_9 = 1.319,<br>P = 0.2198			5 mice/group
S1. g–CA3	Two-tailed unpaired t test	condition	t_9 = 1.141,<br>P = 0.2833			5 mice/group
S1. g–CA1	Two-tailed unpaired t test	condition	t_9 = 2.150,<br>P = 0.0601			5 mice/group
S1. g–HC	Two-tailed unpaired t test	condition	t_9 = 1.778,<br>P = 0.1091			5 mice/group
S.2 a	One-way ANOVA	condition	DG: F (2, 23) = 1.277, P = 0.2978;<br>CA3: F (2, 23) = 6.039, P = 0.0078;<br>HC: F (2, 23) = 3.560, P = 0.0450	Tukey's test	CA3: Ctl vs. Iso: P = 0.3223<br>Ctl vs. LY+Iso: P = 0.0527<br>Iso vs. LY+ Iso: P = 0.0061;<br>HC: Ctl vs. Iso: P = 0.1846<br>Ctl vs. LY+Iso: P = 0.4471<br>Iso vs. LY+ Iso: P = 0.0384	Ctl: n = 13 mice<br>Iso: n = 7 mice<br>LY+Iso: n = 6 mice
S.2 b	One-way ANOVA	condition	DG: F (2, 21) = 0.3066, P = 0.7392;	Tukey's test	CA3: Ctl vs. Sevo: P = 0.1400<br>Ctl vs. LY+ Sevo: P = 0.0322	Ctl: n = 12 mice<br>Sevo: n = 6 mice<br>LY+ Sevo: n = 6 mice

			CA3: F (2, 21) = 8.454, P = 0.0020;<br>HC: F (2, 21) = 3.523, P = 0.0480		Sevo vs. LY+ Sevo: P = 0.0015;<br>HC: Ctl vs. Sevo: P = 0.1702<br>Ctl vs. LY+ Sevo: P = 0.4985<br>Sevo vs. LY+ Sevo: P = 0.0410	
S.2 c	One-way ANOVA	condition	DG: F (2, 21) = 0.4373, P = 0.6516;<br>CA3: F (2, 21) = 0.6291, P = 0.5428;<br>HC: F (2, 21) = 1.033, P = 0.3733			Ctl: n = 12 mice<br>Iso: n = 6 mice<br>LY+Iso: n = 6 mice
S.2 d	One-way ANOVA	condition	DG: F (2, 23) = 0.8988, P = 0.4209;<br>CA3: F (2, 23) = 4.960, P = 0.0162;<br>HC: F (2, 23) = 3.966, P = 0.0331	Tukey's test	CA3: Ctl vs. Sevo: P = 0.1992<br>Ctl vs. LY+ Sevo: P = 0.0153<br>Sevo vs. LY+ Sevo: P = 0.4643;<br>HC: Ctl vs. Sevo: P = 0.0522<br>Ctl vs. LY+ Sevo: P = 0.1165<br>Sevo vs. LY+ Sevo: P = 0.9666	Ctl: n = 13 mice<br>Sevo: n = 7 mice<br>LY+ Sevo: n = 6 mice
S.3 b	Two-tailed Mann-Whitney test	condition	U = 9,<br>P = 0.1014			Ctl: n = 7 mice;<br>KD: n = 6 mice
S.3 c	Two-tailed t test	condition	t_{11} = 0.4397,<br>P = 0.6687			
S.3 d	Two-tailed t test	condition	t_{11} = 1.242,<br>P = 0.2399			
S.4 d	One-way ANOVA	condition	F (2, 15) = 0.8086,<br>P = 0.4640			6 mice/group

S.4 e	One-way ANOVA	condition	F (2, 15) = 5.800, P = 0.0136	Tukey's test	Ctl vs. Sevo: P = 0.3210; Ctl vs. LY+Sevo: P = 0.0104; Sevo vs. LY+Sevo: P = 0.1721;	6 mice/group
S.4 f	One-way ANOVA	condition	F (2, 15) = 3.054, P = 0.0771			6 mice/group
